# Cannulated, locking blade plates for proximal femoral osteotomy in children and adolescents

**DOI:** 10.1007/s11832-015-0649-9

**Published:** 2015-03-24

**Authors:** Leena Zhou, Mark Camp, Abhay Gahukamble, Abhay Khot, H. Kerr Graham

**Affiliations:** 1 Department of Paediatrics, The University of Melbourne, Carlton, VIC 3052 Australia; 2 Department of Orthopaedics, The Royal Children’s Hospital, Flemington Road, Parkville, VIC 3052 Australia; 3 Murdoch Childrens Research Institute, Flemington Road, Parkville, VIC 3052 Australia

**Keywords:** Proximal femoral osteotomy, Cannulated blade plate, Locking screw fixation

## Abstract

**Background:**

Proximal femoral osteotomy is the most common major reconstructive surgery in the region of the hip joint in children and adolescents. Given that it may be required across a wide range of ages and indications, appropriate instrumentation is necessary to ensure a technically satisfactory result. Recent developments in fixation include cannulation of the blade plate and locking screw technology.

**Methods:**

We conducted a prospective audit of our first 25 patients who had a unilateral or bilateral proximal femoral osteotomy using a recently available system which combines cannulation and locking plate technology. The principal outcome measures were the radiographic position of the osteotomy at the time of union and surgical adverse events.

**Results:**

Forty-five proximal femoral osteotomies were performed in 25 patients, mean age 8 years (range 3–17 years), for a variety of indications, the most common of which was hip subluxation in children with cerebral palsy. All osteotomies were soundly united by 6 weeks in children and by 3 months in adolescents, in the position achieved intra-operatively. There were no revision procedures and the technical goals of surgery were achieved in all patients. There was one adverse event, a low-grade peri-prosthetic infection, diagnosed at the time of implant removal.

**Conclusions:**

In this prospective audit of our first 25 patients, the new system performed well across a wide range of ages, body weights and surgical indications. Further comparative studies will be required to determine whether it offers additional advantages over more traditional systems.

## Background

Proximal femoral osteotomy (PFO) is the most common major reconstructive surgery in the region of the hip in children and adolescents [1]. The indications are wide and include developmental dysplasia of the hip (DDH), containment in Perthes’ disease, realignment of the proximal femur following slipped capital femoral epiphysis and stabilisation of the hip in a wide range of neuromuscular diseases, the most common of which is cerebral palsy (CP) [2–5]. Implants for proximal femoral osteotomy may be required for internal fixation from approximately the age of 12 months to skeletal maturity and across a range of body weights, typically ranging from 10 kg to more than 120 kg. Historically, two-part devices were first used in proximal femoral osteotomy in children, derived from devices used for the fixation of proximal femoral fractures in adults [6]. With time, fixed-angle blade plates became the most widely used implant because of their sound biomechanical basis supported by several large retrospective studies showing excellent results [7, 8].

Recent developments in fracture fixation, incorporating locking screws to improve the biomechanical construct, have been introduced to implants for internal fixation in proximal femoral osteotomy [9–11]. A second significant innovation was the development of a one-piece cannulated blade plate, which can be inserted over a guide wire [12]. The guide wire is inserted in the desired position in the proximal femur. The seating chisel is passed over the guide wire to cut the track for the blade plate, which is also inserted over the guide wire. This differs from classical PFO with AO blade plate fixation, in which a guide wire is inserted in the proximal femur and the insertion of the seating chisel and implant is referenced from the position and direction of this wire, but not passed directly over the guide wire [7, 8]. The cannulated blade plate concept was described by Grant and colleagues in 1990 but has only become widely available commercially in recent years [12, 13]. In 2013, Poul and colleagues reported more precise correction of proximal femoral deformities in a study in which the Cannulated Paediatric Osteotomy System (CAPOS) was compared to the conventional angled blade plate (Synthes) [13].

Approximately 160 proximal femoral osteotomies per annum are performed at our tertiary-level paediatric hospital. The safety and efficacy of this procedure is therefore very important in our overall service. It is also important for us to be able to teach this surgery to our surgical registrars and fellows. We hypothesised that this new system would offer significant advantages over existing systems, including more stable fixation and an easier and safer learning curve for trainees. We decided to introduce the implant in a controlled fashion and prospectively audit our first 25 patients. Our study goals were limited to short-term, technical outcomes of the PFO. Given the diverse diagnoses, clinical and functional outcomes would require long-term follow-up of larger, homogeneous subgroups.

An Excel (Microsoft) spreadsheet was devised to include demographic details, diagnosis, surgical indications, surgical planning, operation report, the implant used, pre- and post-operative radiographic data and surgical adverse events. No outside funding was received in relation to this study. The study was conducted under the audit provisions of the hospital’s research and ethics committee.

## Materials and methods

This was a prospective cohort study, with consecutive enrolment of all children and adolescents who had a PFO with the PediLoc^®^ Locking Cannulated Blade Plate System (OrthoPediatrics), between April 1, 2013 and July 31, 2014 (Table 1). Patient data was entered on an Excel spreadsheet from the patient’s electronic scanned medical record (ESMR), and operation reports and radiological data from the Patient Archiving and Communication System (PACS). Pre-operative radiographs were reviewed on Synapse (Fujifilm, PACS) and a number of radiographic parameters measured, relevant to the underlying diagnosis. These included migration percentage (MP), neck shaft angle (NSA) and trochanteric height. Neck shaft angle was measured from an AP radiograph with the hips internally rotated by an amount equal to the estimated femoral neck anteversion (FNA), usually about 40° [14]. FNA was measured by the trochanteric prominence test (TPAT) and confirmed by computed tomography in selected patients [15]. Ambulant children with CP had three-dimensional gait analysis to plan single event, multilevel surgery (SEMLS) [16, 17].

**Table 1:** Diagnosis, surgical indication, operative procedure and outcome of 25 patients with proximal femoral osteotomy and cannulated locking blade plate fixation

ID	Diagnosis	GMFCS level	Indication	Operation	Outcome
1	CP	V	Subluxation	Bilateral VDRO	S
2	CP	V	Subluxation	Bilateral VDRO	S
3	CP	V	Subluxation	Bilateral VDRO	S
4	CP	V	Subluxation	Bilateral VDRO	S
5	NM	IV	Windswept hips	Bilateral VDRO	S
6	CP	II	SEMLS	Bilateral FDO	Infection
7	CP	V	Subluxation	Bilateral VDRO	S
8	CP	V	Subluxation	Bilateral VDRO	S
9	CP	V	Subluxation	Bilateral VDRO	S
10	CP	II	SEMLS	Bilateral FDO	S
11	LCP	TD	Coxa vara	Right valgus PFO	S
12	CP	V	Dislocation	Bilateral VDRO	S
13	CP	IV	Subluxation	Bilateral VDRO	S
14	CP	V	Subluxation	Bilateral VDRO	S
15	Metabolic	V	Fracture	Left hip fracture ORIF	S
16	NM	V	Subluxation	Bilateral VDRO	S
17	CP	III	SEMLS	Bilateral FDO	S
18	CP	IV	Subluxation	Bilateral VDRO	S
19	CP	IV	Subluxation	Bilateral VDRO	S
20	CP	II	SEMLS	Bilateral FDO	S
21	DDH	TD	Subluxation	Left VDRO	S
22	LCP	TD	Containment	Right VDREO	S
23	DDH	TD	Dislocation	OR/left VDRO	Unknown
24	Metabolic	IV	Subluxation	Bilateral VDRO	S
25	CP	II	SEMLS	Bilateral FDO	S

*CP* cerebral palsy, *NM* neuromuscular, *LCP* Legg–Calvé–Perthes disease, *DDH* developmental dysplasia of the hip, *GMFCS* gross motor function classification system, *TD* typically developing, *VDRO* varus derotation osteotomy, *FDO* femoral derotation osteotomy, *PFO* proximal femoral osteotomy, *OR* open reduction, *ORIF* open reduction internal fixation, *VDREO* varus derotation extension osteotomy, *S* satisfactory: defined as no change in the position of the implant between the time of surgery and latest follow-up, union within 6 weeks in children and 12 weeks in adolescents, and all technical goals of surgery achieved

Sequential radiographs were obtained before discharge from hospital and at 3, 6 and 12 weeks after surgery. For the purposes of this study, bony union was defined as the presence of bridging callus across >75 % of the osteotomy surface as well as external, medial bridging callus. The time to union was noted and the position of the implants studied on PACS at each interval, specifically looking for signs of union and any change in position of the implant or osteotomy. Technical outcomes of the surgery including femoral head cover (MP) and change in neck shaft angle were also observed. Changes in MP and NSA in the non-ambulant CP [gross motor function classification system (GMFCS) IV and V] subgroup were compared using paired *t* tests with *P* < 0.05.

### Surgical technique

PFOs were performed under general anaesthesia, peri-operative intravenous antibiotics and insertion of an epidural catheter and bladder catheter when considered appropriate [16, 18]. The patient was placed supine on a radiolucent operating table. The lower limbs were prepared and draped free and a preliminary fluoroscopic examination was performed to confirm adequate range of motion, that any subluxation of the femoral head was reducible and that the goals of surgery were technically feasible [18]. The need for additional soft tissue releases and bony procedures was also assessed, including adductor and psoas releases, open reduction of the hip and pelvic osteotomy [16–19]. Biplanar fluoroscopy was used throughout the procedure with the C-arm in a fixed position over the operated hip. The lateral radiographs were obtained by moving the operated limb into a flexed, abducted position [19].

**Fig. 1:**
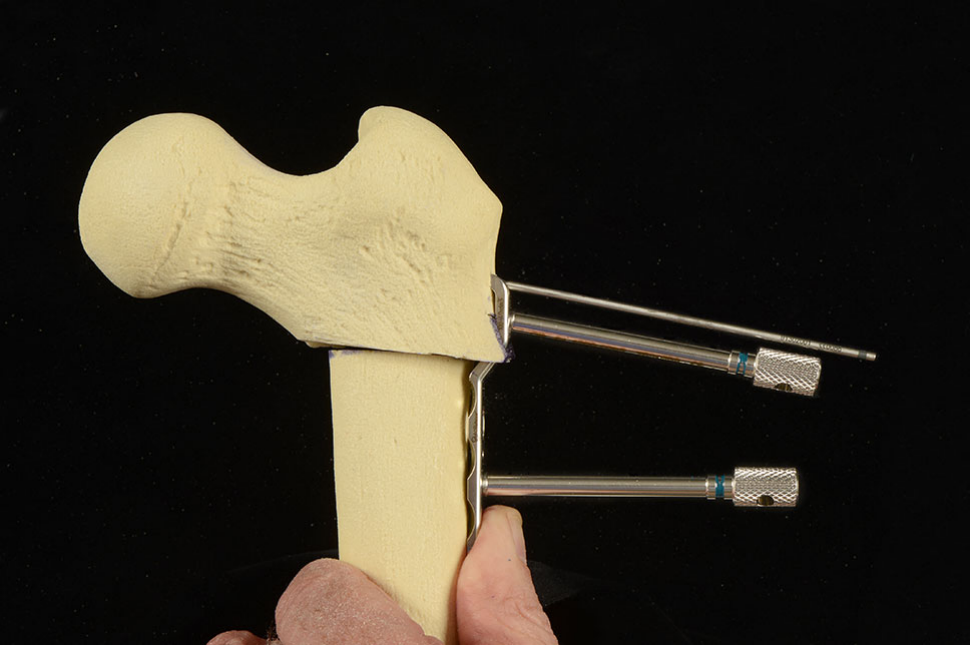
Cannulated locking blade plate fixation in a sawbone model of VDRO of the proximal femur. Note that the blade plate has been inserted over the guide wire. Two locking towers are in position, in the first screwhole (which is inserted into the proximal metaphysis) and the third screwhole (which is inserted into the diaphysis). The remaining screwholes, the second and fourth, are for non-locking screws and offer the opportunity for compression prior to the insertion of the locking screws

**Fig. 2:**
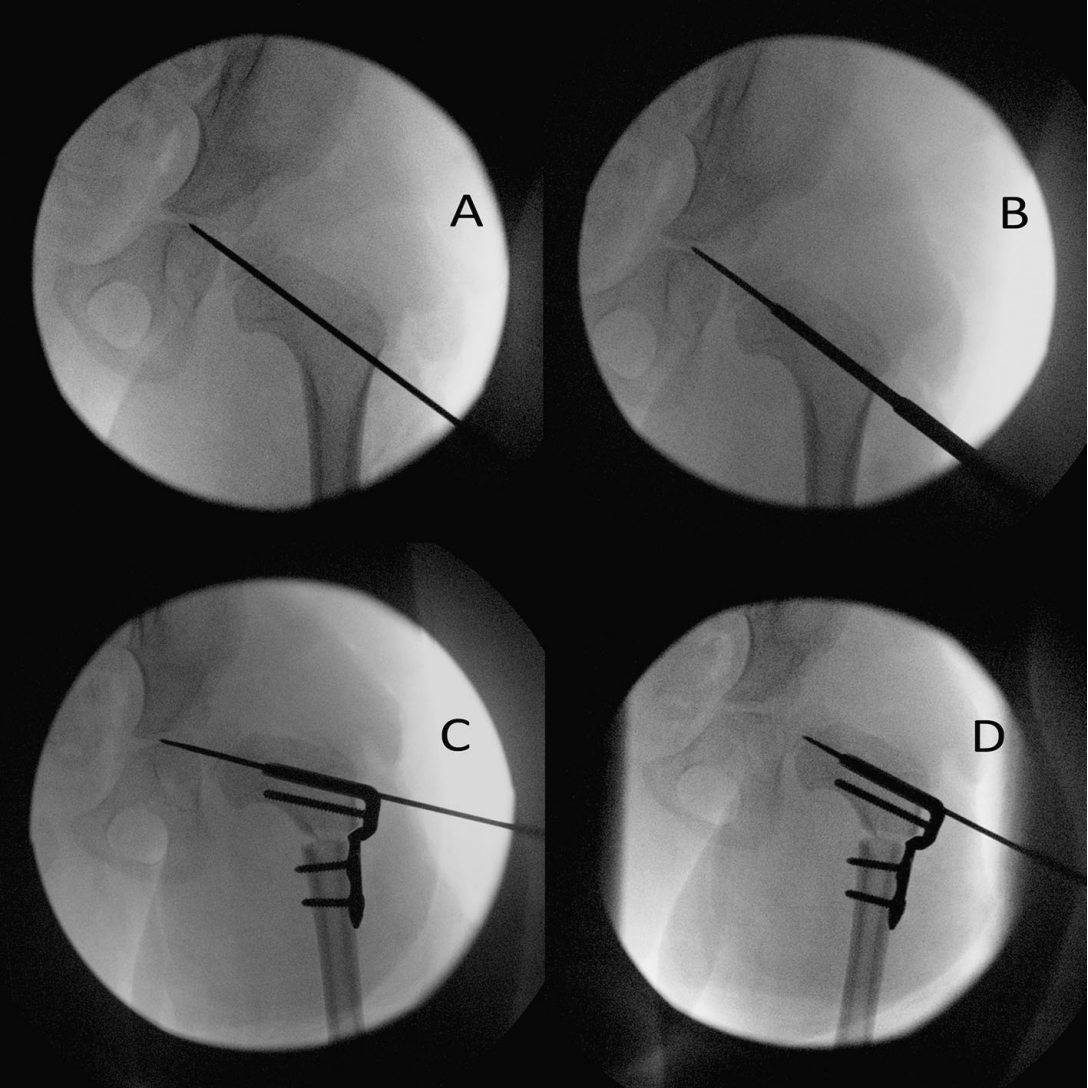
Four fluoroscopic images showing the sequence of fixation using the cannulated locking blade plate. **a** Revision anterolateral open reduction has been performed for left DDH. The guide wire has been inserted close to the centre of the proximal femoral metaphysis and advanced across the proximal femoral growth plate and into the triradiate cartilage to stabilise the open reduction. NB: This is not a standard step. **b** The seating chisel has been advanced across the guide wire. **c** The 90° toddler plate has been bent to change the NSA to 100°. The osteotomy and shortening have been performed and the blade plate has been inserted and fixation screws placed. **d** The guide wire has been partially removed to check the stability of the open reduction and the stability of the VDRO. Following this, the guide wire was removed and a hip spica cast applied, the only hip spica in the series

The proximal femur was exposed subperiosteally with careful retraction of the vastus lateralis to preserve its nerve and blood supply. A suitable entry for the insertion of the guide wire was marked using a combination of analysis of the surgical goals, the likely implant to be used and the patient’s specific anatomy. For the most common procedure (varus derotation osteotomy in CP) the goal was a NSA of 100° and complete containment of the femoral head. The guide wire was inserted into the centre of the femoral neck on both AP and lateral radiographic projections and a 100° plate of the appropriate width and length was selected. Selection of the blade width was aided by passing a seating chisel over the guide wire and, in the flexed-abducted view, checking fluoroscopically that the chisel (and plate) would be able to cross the isthmus of the neck without risk of penetration or fracture. The appropriate seating chisel was used to cut a track for the selected blade plate; the seating chisel and blade plate were matched. Attention was given to cutting the track collinear with the guide wire to avoid inadvertent advancement or removal of the guide wire. The seating chisel was then backed out slightly but left in situ [12]. The appropriate osteotomy was then marked and performed using an oscillating saw with the appropriate spacing device to make sure that the cut was at an appropriate distance from the seating chisel. The first cut was made parallel to the seating chisel. Depending on the correction desired, the second cut was made on the distal fragment to improve the reduction of the osteotomy and coaptation of the fragments [18, 19]. If there was a significant medial offset, then a third cut was made on the lateral surface of the proximal fragment to recess the plate against the femur. This had the added advantages of increasing proximal stability and preventing implant prominence and soft tissue irritation. The osteotomy surfaces were coapted to check for the fit. The seating chisel was removed and the blade plate inserted over the guide wire. A reduction clamp was then used to finalise the position of the implant with respect to both parts of the proximal femur. Preliminary fixation was performed using a non-locking screw to achieve compression at the osteotomy site. Fixation was then completed using the requisite number of locking and non-locking screws (Fig. 1). The position of the implant was recorded in both AP and lateral fluoroscopic projections. The hip was put through a range of motion to ensure that fixation was stable. The fluoroscopic images were also scrutinised to determine whether the goals of surgery had been achieved: e.g., femoral head cover. The stability of fixation was assessed and this determined the need for hip spica casting, restricted weight-bearing or immediate mobilisation (Fig. 2). The guide wire was removed and, following irrigation, the incision was closed in layers. Routine post-operative care was provided with formal imaging of the osteotomy prior to discharge from hospital. Immediate range of motion, physiotherapy and full weight-bearing as tolerated was encouraged in all patients apart from one 3-year-old girl with DDH who had a revision open reduction and capsulorraphy.

All patients were followed closely after surgery to monitor healing, function and surgical adverse events (SAEs). SAEs were classified according to the modified Clavien–Dindo system [20].

Descriptive statistics were obtained from Excel, including means and standard deviations (SDs). Statistical analyses were performed using Stata Statistical Software, release 13 (StataCorp, College Station, Texas, USA).

## Results

Between April 1, 2013 and July 31, 2014, 45 proximal femoral osteotomies were performed in 25 children and adolescents. The majority of PFOs were performed by registrars and fellows, under the supervision of the senior authors. There were 13 boys and 12 girls with a mean age of 7 years and 9 months (range of 3–17 years) and a mean weight of 22.7 kg (range 11–66 kg).

Mean follow-up was 9 months (SD range 4–20 months). All children were followed to bony union apart from one girl with DDH who moved to another state, within 4 weeks of surgery, whilst in hip spica cast.

**Fig. 3:**
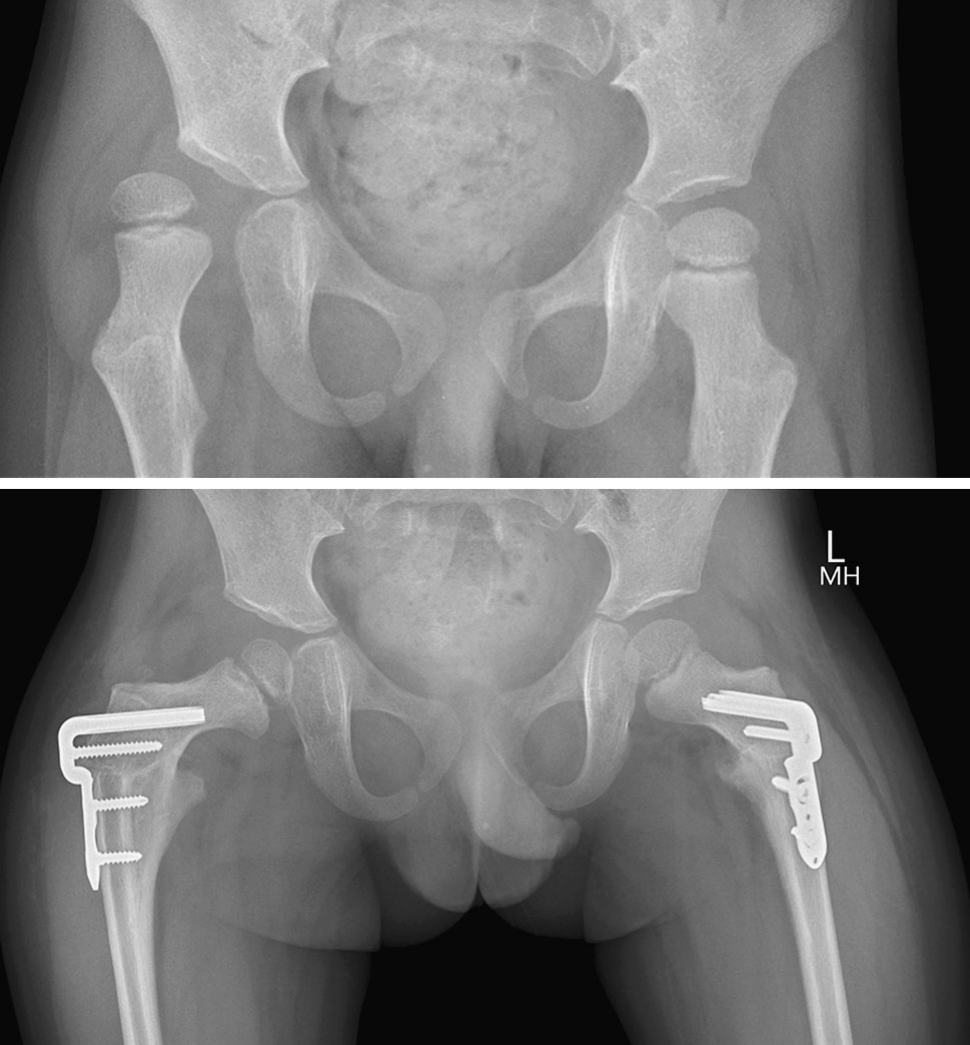
Right hip dislocation in a 5-year-old boy with severe cerebral palsy and poor nutritional status, weighing 12 kg. The right hip was already painful. Bilateral VDROs with the infant plate showing containment of both hips, sound bony union with no loss of position and no requirement for a hip spica cast

**Fig. 4:**
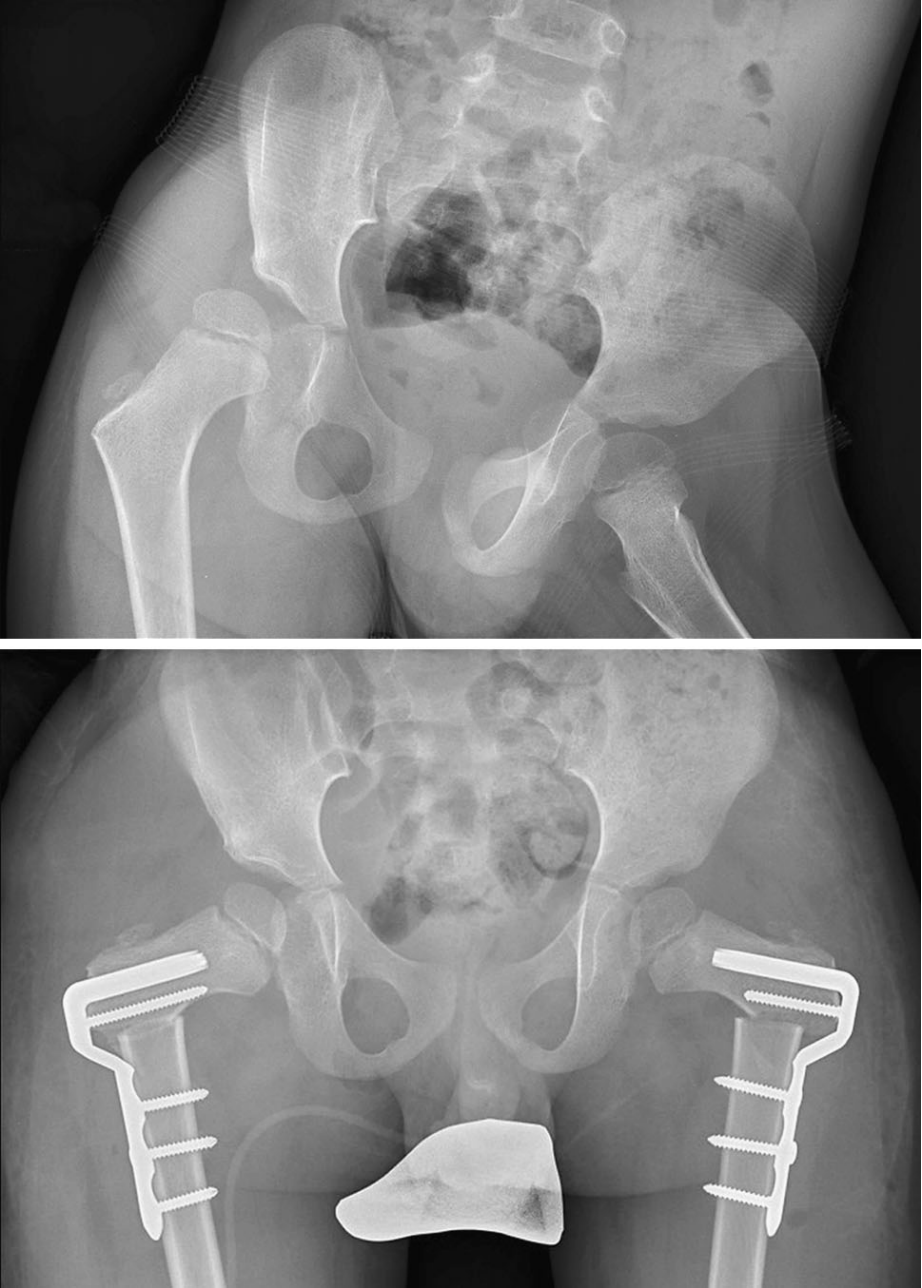
Windswept hips in a 7-year-old boy with a neuromuscular disease. A left hip abduction contracture and right hip adduction contracture were released and combined with bilateral VDROs using 40 mm, 90° child plates. Both hips are contained and the pelvis has been levelled. This is the simplest type of osteotomy because the guide wire, chisel and blade plate are placed centrally in the proximal metaphysis

**Fig. 5:**
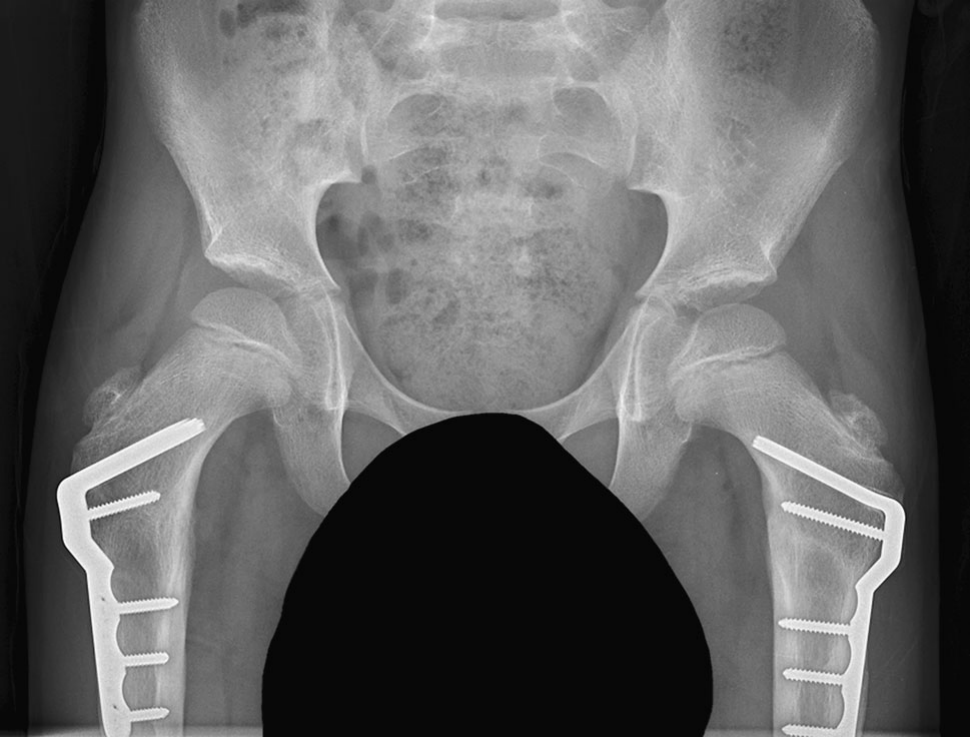
Bilateral FDOs with the 100° cannulated locking blade plate, in a 10-year old boy with cerebral palsy, GMFCS level II, undergoing SEMLS. The goal was a 40° external rotation correction on both sides with no change in NSA. Note the position of the tip of the blade in the strong bone of the calcar and inferior femoral neck. The patient mobilised full weight-bearing within a week of surgery and had excellent correction of internal rotation gait

Seventeen children had cerebral palsy, two had other neuromuscular diseases, two had DDH, two had metabolic disease and two had Perthes’ disease. Five ambulant children with CP had bilateral external rotation PFOs to improve gait, 15 children with CP/other neuromuscular and metabolic disorders had bilateral varus derotation osteotomies (VDROs) for hip subluxation, two girls with DDH had unilateral VDROs, one boy with Perthes’ disease had unilateral VDRO for containment and another boy with Perthes’ disease had valgus osteotomy for coxa vara. One child with a metabolic disorder had fixation of an insufficiency fracture. In the CP/neuromuscular group who had bilateral VDROs for hip subluxation (Fig. 3), the mean MP pre-operatively was 48 % (SD 24 %) and post-operatively was 11 % (SD 13 %) The mean NSA was 155° (SD 9.6°) and the mean post-operative NSA was 112° (SD 9°). These changes were significant at *P* < 0.001. The ambulant CP group had satisfactory correction of internal rotation gait clinically, which was confirmed on gait analysis in the first two patients who have had follow-up gait analysis (Figs. 4, 5).

In the two girls with DDH, one achieved correction of long leg dysplasia by a PFO which included 30° derotation, 10° varus and 1 cm of shortening. In the other girl, VDRO was used to stabilise a revision open reduction with varus of 20°, derotation of 40° and shortening of 1 cm (Fig. 2).

**Fig. 6:**
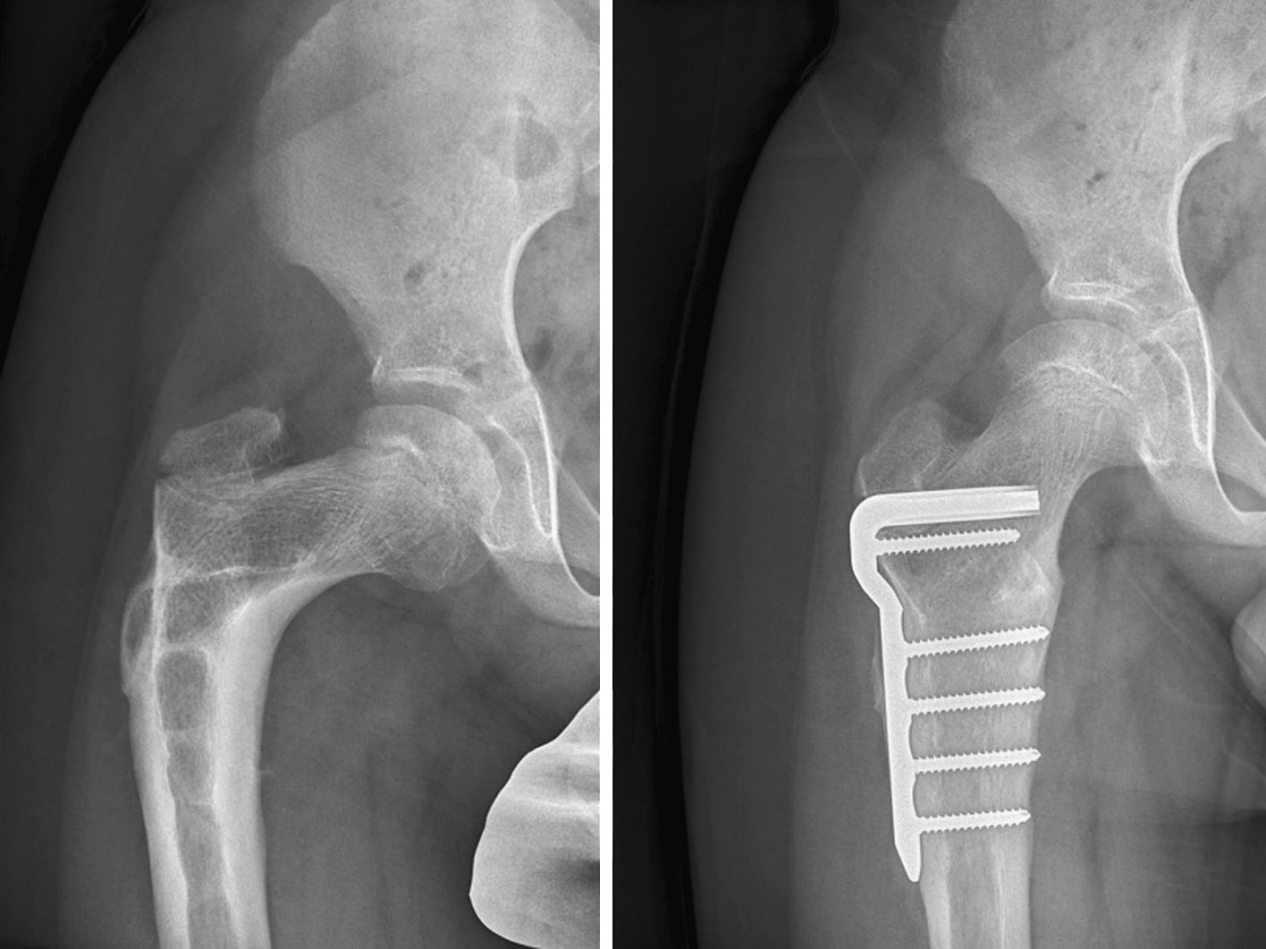
Iatrogenic coxa vara in a 10-year-old boy previously treated by VDRO for Perthes’ disease. He presented with fatigue pain and limping due to abductor insufficiency. The preoperative NSA was 94°. A valgus proximal femoral osteotomy was formed with a 90° plate and the NSA was restored to 132°, with correction of limb length discrepancy and the abductor insufficiency. A pelvic osteotomy will be required in due course

The boy with Perthes’ disease had satisfactory containment following varus of 15°, extension of 15° and external rotation of 20°. The second boy with Perthes’ disease had satisfactory correction of iatrogenic coxa vara by valgus PFO of 30°. His mean pre-operative NSA of 94° was corrected to 134° post-operatively. (Fig. 6).

There were no delayed unions, mal-unions or loss of position. There were no revision procedures for implant-related complications. One boy with neuromuscular disease and impaired immunity had a low-grade *Staphylococcus epidermidis* infection. The infection was asymptomatic and was only recognised at the time of routine plate removal by the culture of a small amount of fluid which was present around the blade of the implant. The infection was eradicated following removal of the implant and antibiotics. This was a Grade III complication, according to the modified Clavien–Dindo system [20].

## Discussion

Proximal femoral osteotomy is an important reconstructive option in the management of a wide range of hip pathology in children and adolescents [1–8]. The implants for this procedure have paralleled advances in implant technology for fixation of proximal femoral fractures and osteotomies in adults [6–11]. More recently, developments in cannulated device technology along with locking plate technology have been combined into a new system, which appears to have the versatility to cover the full range of reconstructive requirements from birth to skeletal maturity in children’s orthopaedics [12]. The principles and surgical approach are built solidly on the foundations of previous technology and require only minor modifications. We conducted an audit of our first 25 patients to determine the complication rate during the learning curve as well as the ability of established and trainee surgeons to learn how to use the new system. Technically, the system performed faultlessly with no loss or change of position between the insertion of implants and bony union at 6–12 weeks after surgery. The stability of fixation was felt to be adequate, even in younger children with CP who had hypertonia, nutritional impairment and osteopenia [19]. Apart from one girl with an open reduction of DDH, hip spicas were not used. No restrictions were placed on mobility or weight-bearing, which was a major advantage for early rehabilitation after SEMLS [16]. Despite this immediate mobilisation policy, there were no changes in implant position, and the technical goals of all procedures were achieved. This zero revision rate compares favourably with both older and newer systems such as the Richards intermediate hip screw, the AO blade plate and the Pediatric LCP Plate [7, 8, 10, 11]. In addition, older systems were more likely to be followed by hip spica casting, which has high costs in terms of time and materials, is inconvenient for families and is associated with significant morbidity [8, 10, 17, 18, 21, 22]. The LCP system also employs a locking screw technology but there are significant restrictions on the choice of NSA, given that most commercial systems have only two side-plate angles for varus correction [11, 12]. In addition, delayed union seems to be a problem with the LCP system, where the fixation system seems to be too rigid for some paediatric indications [10, 11].

The limitations of our study include the wide range of ages and indications, the concentration on purely technical and not functional outcomes and the small number of patients and osteotomies. Future studies will include comparison of this device with existing technology. In addition, a detailed study of attitudes and comfort levels from trainees at different levels will be conducted again in comparison with existing non-cannulated, non-locking technology.
